# Supplementary material for: Molecular-Biology-Driven Frontline Treatment for Chronic Lymphocytic Leukemia: A Network Meta-Analysis of Randomized Clinical Trials
Source: Int J Mol Sci. 2023 Jun 9;24(12):9930. doi: 10.3390/ijms24129930 (PMC10298034; doi:10.3390/ijms24129930)
Supplement: Supplementary file 1 [file ijms-24-09930-s001.zip › ijms-2270156-supplementary.pptx]

## Slide 1
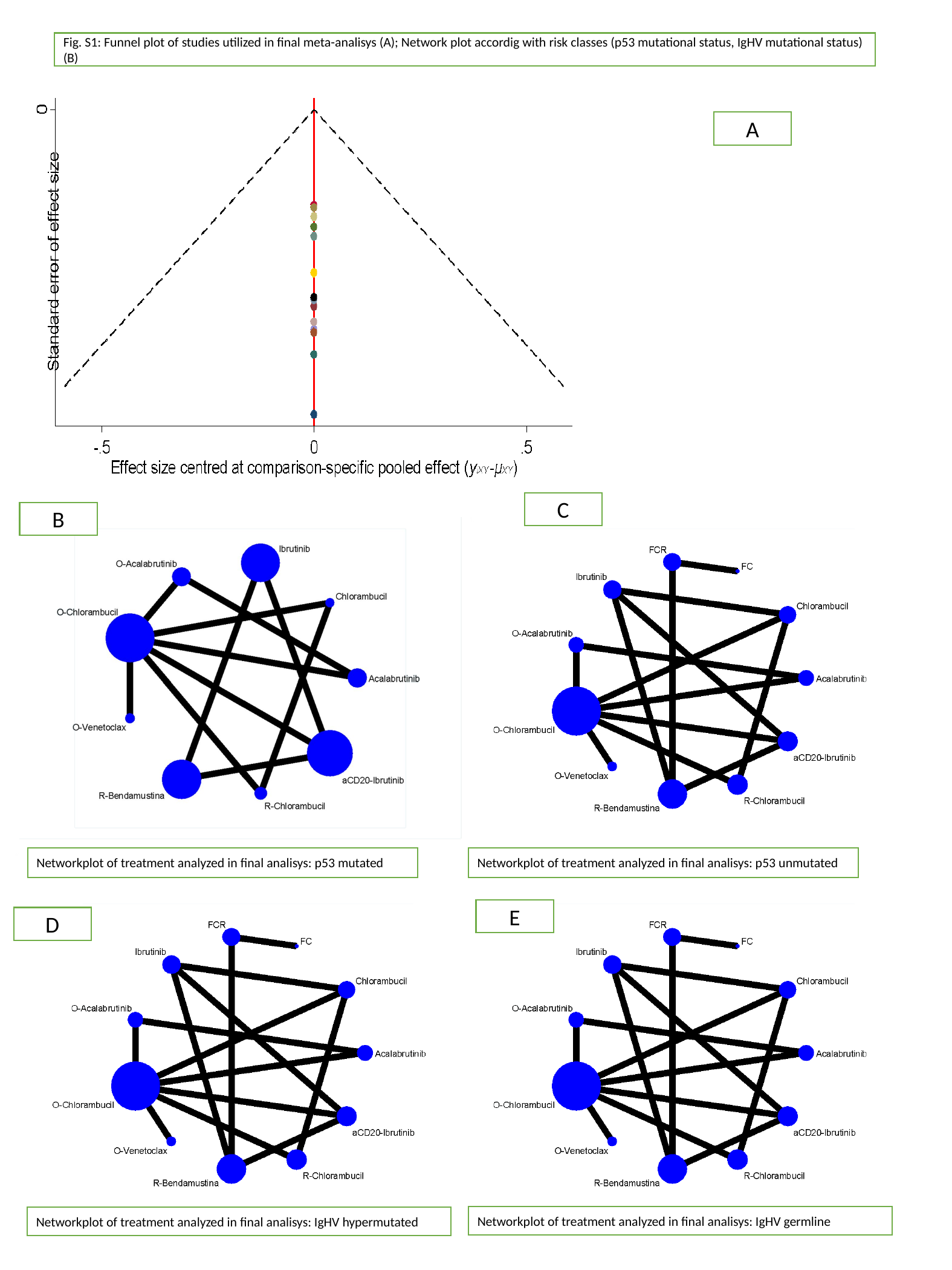

Fig. S1: Funnel plot of studies utilized in final meta-analisys (A); Network plot accordig with risk classes (p53 mutational status, IgHV mutational status) (B)
A
C
B
Networkplot of treatment analyzed in final analisys: p53 mutated
Networkplot of treatment analyzed in final analisys: p53 unmutated
E
D
Networkplot of treatment analyzed in final analisys: IgHV germline
Networkplot of treatment analyzed in final analisys: IgHV hypermutated

## Slide 2
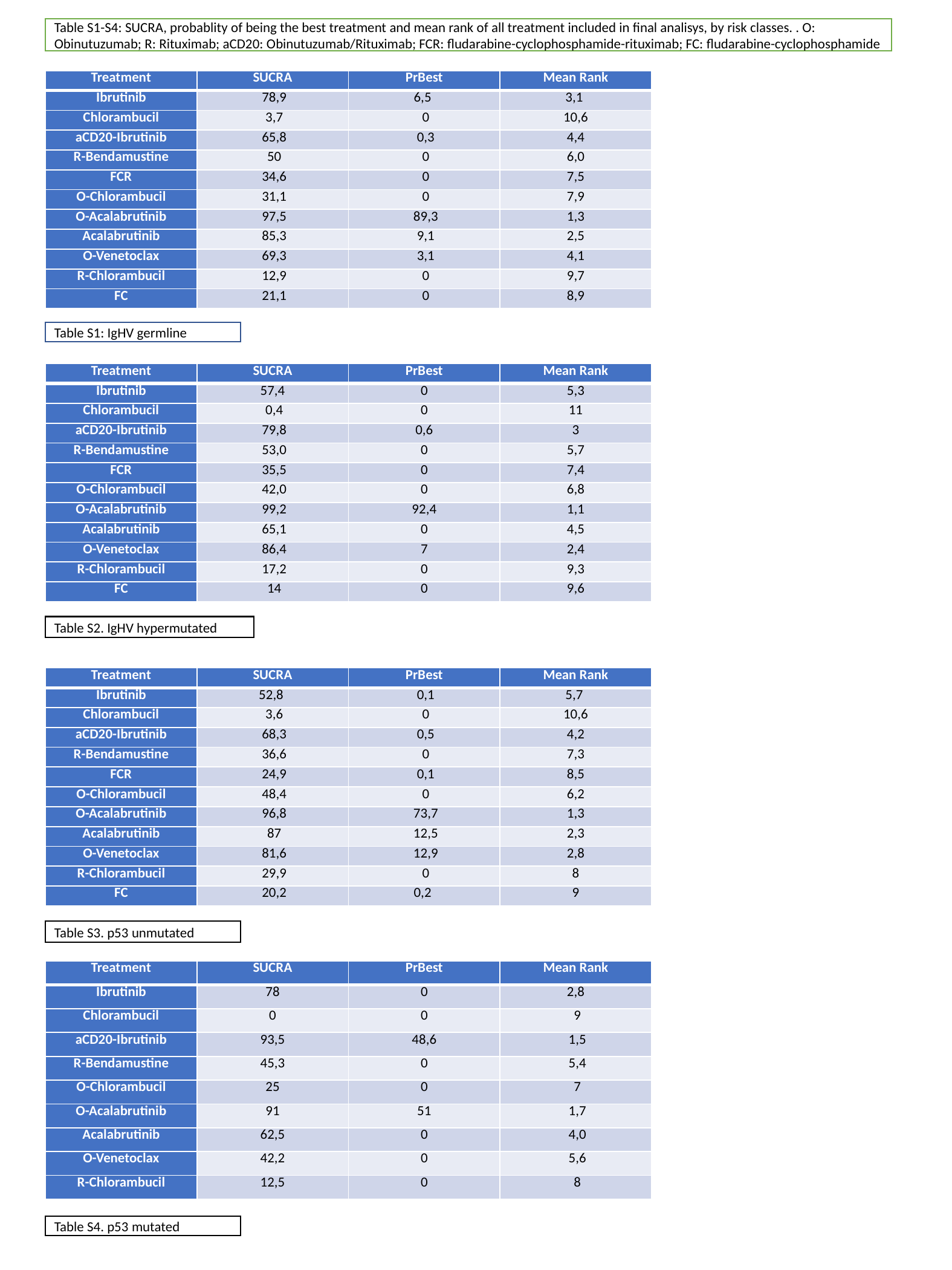

Table S1-S4: SUCRA, probablity of being the best treatment and mean rank of all treatment included in final analisys, by risk classes. . O: Obinutuzumab; R: Rituximab; aCD20: Obinutuzumab/Rituximab; FCR: fludarabine-cyclophosphamide-rituximab; FC: fludarabine-cyclophosphamide
| Treatment | SUCRA | PrBest | Mean Rank |
| --- | --- | --- | --- |
| Ibrutinib | 78,9 | 6,5 | 3,1 |
| Chlorambucil | 3,7 | 0 | 10,6 |
| aCD20-Ibrutinib | 65,8 | 0,3 | 4,4 |
| R-Bendamustine | 50 | 0 | 6,0 |
| FCR | 34,6 | 0 | 7,5 |
| O-Chlorambucil | 31,1 | 0 | 7,9 |
| O-Acalabrutinib | 97,5 | 89,3 | 1,3 |
| Acalabrutinib | 85,3 | 9,1 | 2,5 |
| O-Venetoclax | 69,3 | 3,1 | 4,1 |
| R-Chlorambucil | 12,9 | 0 | 9,7 |
| FC | 21,1 | 0 | 8,9 |
Table S1: IgHV germline
| Treatment | SUCRA | PrBest | Mean Rank |
| --- | --- | --- | --- |
| Ibrutinib | 57,4 | 0 | 5,3 |
| Chlorambucil | 0,4 | 0 | 11 |
| aCD20-Ibrutinib | 79,8 | 0,6 | 3 |
| R-Bendamustine | 53,0 | 0 | 5,7 |
| FCR | 35,5 | 0 | 7,4 |
| O-Chlorambucil | 42,0 | 0 | 6,8 |
| O-Acalabrutinib | 99,2 | 92,4 | 1,1 |
| Acalabrutinib | 65,1 | 0 | 4,5 |
| O-Venetoclax | 86,4 | 7 | 2,4 |
| R-Chlorambucil | 17,2 | 0 | 9,3 |
| FC | 14 | 0 | 9,6 |
Table S2. IgHV hypermutated
| Treatment | SUCRA | PrBest | Mean Rank |
| --- | --- | --- | --- |
| Ibrutinib | 52,8 | 0,1 | 5,7 |
| Chlorambucil | 3,6 | 0 | 10,6 |
| aCD20-Ibrutinib | 68,3 | 0,5 | 4,2 |
| R-Bendamustine | 36,6 | 0 | 7,3 |
| FCR | 24,9 | 0,1 | 8,5 |
| O-Chlorambucil | 48,4 | 0 | 6,2 |
| O-Acalabrutinib | 96,8 | 73,7 | 1,3 |
| Acalabrutinib | 87 | 12,5 | 2,3 |
| O-Venetoclax | 81,6 | 12,9 | 2,8 |
| R-Chlorambucil | 29,9 | 0 | 8 |
| FC | 20,2 | 0,2 | 9 |
Table S3. p53 unmutated
| Treatment | SUCRA | PrBest | Mean Rank |
| --- | --- | --- | --- |
| Ibrutinib | 78 | 0 | 2,8 |
| Chlorambucil | 0 | 0 | 9 |
| aCD20-Ibrutinib | 93,5 | 48,6 | 1,5 |
| R-Bendamustine | 45,3 | 0 | 5,4 |
| O-Chlorambucil | 25 | 0 | 7 |
| O-Acalabrutinib | 91 | 51 | 1,7 |
| Acalabrutinib | 62,5 | 0 | 4,0 |
| O-Venetoclax | 42,2 | 0 | 5,6 |
| R-Chlorambucil | 12,5 | 0 | 8 |
Table S4. p53 mutated

## Slide 3
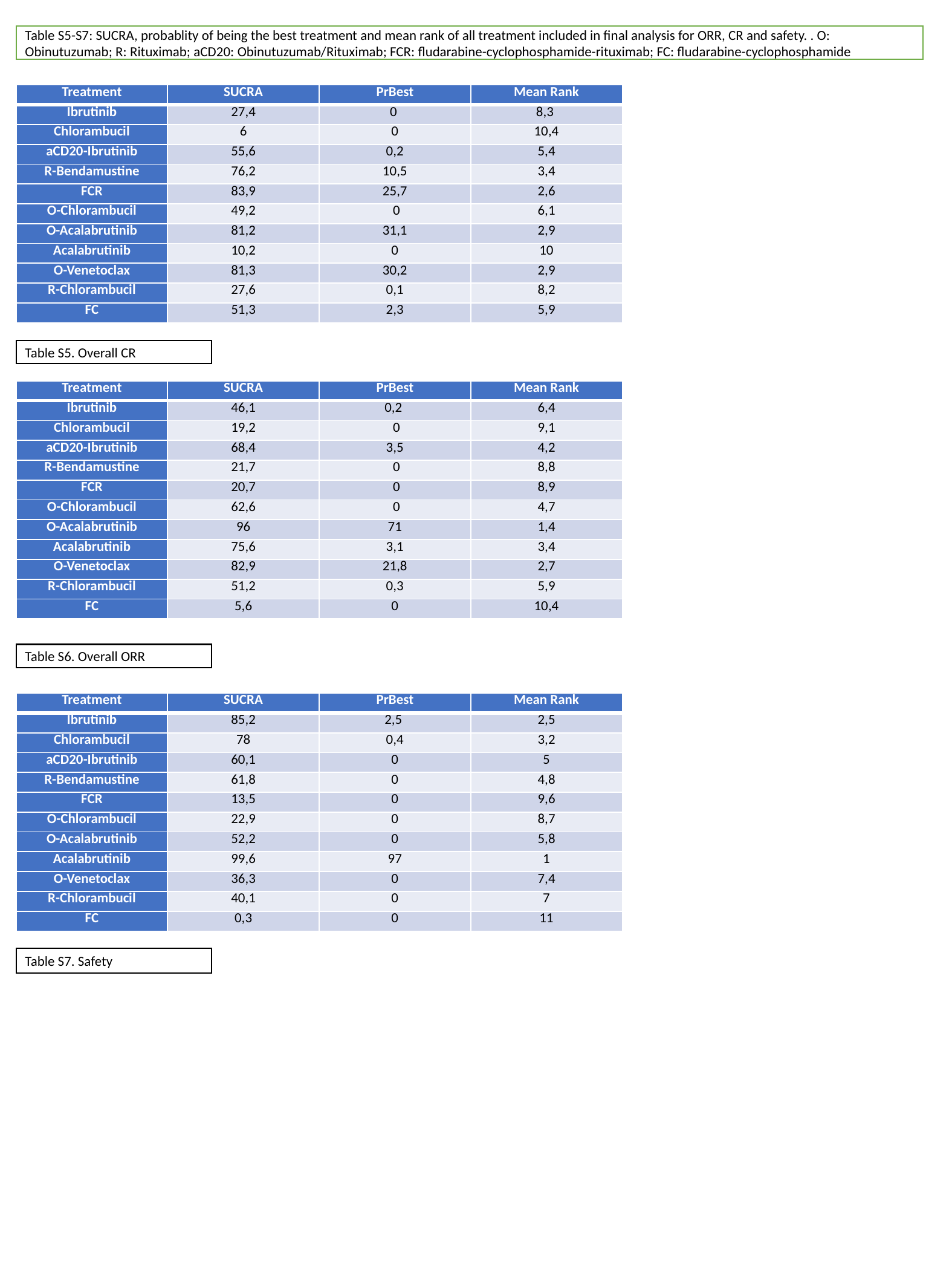

Table S5-S7: SUCRA, probablity of being the best treatment and mean rank of all treatment included in final analysis for ORR, CR and safety. . O: Obinutuzumab; R: Rituximab; aCD20: Obinutuzumab/Rituximab; FCR: fludarabine-cyclophosphamide-rituximab; FC: fludarabine-cyclophosphamide
| Treatment | SUCRA | PrBest | Mean Rank |
| --- | --- | --- | --- |
| Ibrutinib | 27,4 | 0 | 8,3 |
| Chlorambucil | 6 | 0 | 10,4 |
| aCD20-Ibrutinib | 55,6 | 0,2 | 5,4 |
| R-Bendamustine | 76,2 | 10,5 | 3,4 |
| FCR | 83,9 | 25,7 | 2,6 |
| O-Chlorambucil | 49,2 | 0 | 6,1 |
| O-Acalabrutinib | 81,2 | 31,1 | 2,9 |
| Acalabrutinib | 10,2 | 0 | 10 |
| O-Venetoclax | 81,3 | 30,2 | 2,9 |
| R-Chlorambucil | 27,6 | 0,1 | 8,2 |
| FC | 51,3 | 2,3 | 5,9 |
Table S5. Overall CR
| Treatment | SUCRA | PrBest | Mean Rank |
| --- | --- | --- | --- |
| Ibrutinib | 46,1 | 0,2 | 6,4 |
| Chlorambucil | 19,2 | 0 | 9,1 |
| aCD20-Ibrutinib | 68,4 | 3,5 | 4,2 |
| R-Bendamustine | 21,7 | 0 | 8,8 |
| FCR | 20,7 | 0 | 8,9 |
| O-Chlorambucil | 62,6 | 0 | 4,7 |
| O-Acalabrutinib | 96 | 71 | 1,4 |
| Acalabrutinib | 75,6 | 3,1 | 3,4 |
| O-Venetoclax | 82,9 | 21,8 | 2,7 |
| R-Chlorambucil | 51,2 | 0,3 | 5,9 |
| FC | 5,6 | 0 | 10,4 |
Table S6. Overall ORR
| Treatment | SUCRA | PrBest | Mean Rank |
| --- | --- | --- | --- |
| Ibrutinib | 85,2 | 2,5 | 2,5 |
| Chlorambucil | 78 | 0,4 | 3,2 |
| aCD20-Ibrutinib | 60,1 | 0 | 5 |
| R-Bendamustine | 61,8 | 0 | 4,8 |
| FCR | 13,5 | 0 | 9,6 |
| O-Chlorambucil | 22,9 | 0 | 8,7 |
| O-Acalabrutinib | 52,2 | 0 | 5,8 |
| Acalabrutinib | 99,6 | 97 | 1 |
| O-Venetoclax | 36,3 | 0 | 7,4 |
| R-Chlorambucil | 40,1 | 0 | 7 |
| FC | 0,3 | 0 | 11 |
Table S7. Safety
